# The influence of ambient aroma on middle school students' academic emotions

**DOI:** 10.1002/ijop.12827

**Published:** 2022-01-21

**Authors:** Yuanguang Ma

**Affiliations:** ^1^ Department of Education Binzhou University Binzhou China

**Keywords:** Ambient aroma, Middle school student, Academic emotion

## Abstract

This study investigated the influence of ambient aroma on the academic emotions experienced by middle school students in a practical educational environment. The study was conducted with pre‐ and post‐test experimental design upon subjects in three parallel classes (*n* = 109) in grade two of a junior school in China. These classes were randomly assigned to one of three experimental conditions (no aroma, daily aroma or aroma every other day). The academic emotions of the students were measured twice using the Adolescent Academic Emotion Questionnaire, once before and once 8 weeks after the use of aromatherapy. Comparison of the change in ratings from baseline to post‐test showed that compared to the control class; joy, hope, positive high‐arousal academic emotion and relaxation were significantly higher in ambient aroma conditions, and anger and negative high‐arousal academic emotion in these conditions were significantly lower. The results suggest that the ambient aroma of sweet orange essential oil can mitigate the reduction in positive academic emotion and improve negative academic emotion in school students over time.

Academic emotion refers to a variety of emotional experiences related to students' studies in the learning process, including happiness, calm, anxiety, depression and so on. It includes not only the various emotions experienced by students after learning of their academic success or failure, but also the emotional experiences of students during classroom learning, daily homework or examinations (Dong and Yu, 2007). Studies have shown that academic emotion has an important influence on their mental health and academic achievements (Respondek et al., 2017; Zhu & Zhang, 2017). Others have found that there were many emotional problems, including depression and anxiety among Chinese adolescent students (Wei & Ma, 2011). It is particularly important to determine how to improve their academic emotional state.

Aromatherapy is a psychological therapy that uses aromatic essential oils to have positive effects on the mind, such as reducing depression and anxiety (Hu et al., 2010). Research has shown that aromatic essential oils can reduce depression, anxiety and other negative emotions, and make people feel happier. For example, Lehrner et al. (2000) found that the aroma of orange essential oil was beneficial for relieving the anxiety of patients in a dentist's waiting room. Imura et al. (2006) found that lavender and neroli essential oils could reduce the probability of postpartum depression. Other studies also found that the aroma of lavender could reduce anxiety levels in dental patients (Kritsidima et al., 2010), or in patients in a plastic surgery waiting room (Fenko & Loock, 2014). The subjects in these studies were all adults, so what about the effects of aromas on minors?

Other studies have examined the effects of aromas on minors. For example, Moss et al. (2017) exposed 40 schoolchildren aged nine to 11 to either Rosemary aroma, or no aroma, in a classroom setting where they completed standardised tests of working memory in the independent groups design study. Performances on the working memory tasks were significantly better in the Rosemary aroma condition. Epple and Herz (1999) investigated whether odours can become conditioned to emotionally salient experiences such that when later encountered they influence performance consistent with a previously associated event. To test this hypothesis, 5‐year‐olds were given the experience of failure on a cognitive maze in a room scented with fragrance and later given another cognitively challenging test in a different room scented with either the same odour, a different odour, or no odour. Those who performed the test in the presence of the same odour as the maze task did significantly worse than subjects in any other group. Facial expressions and verbal communications made during the maze task indicated negative affect. Jafarzadeh et al. (2013) investigated the effect of aromatherapy with orange essential oil on the anxiety of children (aged 6–9 years) during dental treatment, and found that it reduced their salivary cortisol level and pulse rate.

To summarise, it was found that studies on the influence of aroma on individual emotions have reached a relatively consistent conclusion: the aroma of essential oils can reduce depression, anxiety or other negative emotions and bring pleasure to people (Mikiko, 2013). However, there are still several problems in the current studies on the influence of aroma on emotion: (a) most of the previous studies have focused on patients, but few have looked at its influence on the general population, especially teenage students; (b) most of the studies have not studied the influence of aroma on academic emotions. Therefore, the aim of this study is to explore the influence of ambient aroma on the academic emotions of middle school students through experimental methods. The research hypotheses are as follows: (a) aroma can improve positive academic emotion; (b) aroma can reduce negative academic emotion; (c) olfactory adaptability will reduce the influence of aroma on academic emotion. In theory, this study will increase our understanding of the influence of aroma on emotion. In practice, it may provide new ideas for schools to create a positive educational environment.

## PRE‐STUDY: SELECT AROMA

The purpose of this study is to evaluate five essential oils; rosemary, lavender, mint, lemon and sweet orange, and select the students' favourite essential oil to prepare for the main study.

## METHODS

### Participants

A total of 45 middle school students from Beizhen middle school were randomly selected, including 22 male students (average age: 13.45 ± 0.96) and 23 female students (average age: 13.52 ± 0.99) with normal intelligence and normal sense of smell. All participants gave signed consent (approved by the local ethics committee) and received a gift after the experiment.

### Materials

The five essential oils (rosemary, lavender, mint, lemon and sweet orange) were sourced from Guangzhou POSHLAM Cosmetics Co., Ltd. and were evaluated on the aspects of pleasantness and relaxation ability using a 5‐point scale.

### Aromatic rating scale

This scale is a 5‐point scale including the two aspects of pleasantness (1 = completely unpleasant to 5 = very pleasant), and relaxation (1 = completely unrelaxing to 5 = completely relaxing) (Fenko & Loock, 2014). Participants were asked to smell scent oil from the bottle and to evaluate the smell on the two aspects using the scales.

### Experimental procedure

The experiment was carried out in a clean, quiet and odourless room. The participants were asked to smell the essential oils in the bottles, and to evaluate their sense of the aromas on the two aspects of pleasantness and relaxation ability. After each smell the participants filled in the table. Before smelling the next fragrance they smelt their own skin to neutralise the previous smell (Fenko & Loock, 2014). The order of the five fragrances was randomly arranged among the participants to eliminate the order effect. At the end of the experiment, the participants were presented with the aromatic name and asked to confirm the previous answer.

## RESULTS

The evaluation results of the five essential oils of 45 participants are shown in Table 1.

**TABLE 1:** Pleasantness and relaxation of five essential oils (*M* ± *SD)*

Type	Pleasantness	Relaxation
Rosemary	2.40 ± 1.03	2.71 ± 1.16
Lavender	2.40 ± 1.03	2.22 ± 1.08
Mint	2.42 ± 1.08	2.62 ± 1.07
Lemon	3.58 ± 1.20	3.49 ± 1.27
Sweet orange	3.84 ± 0.96	3.80 ± 0.97

A repeated‐measures analysis of variance (ANOVA) was conducted to compare the effect of the five different scents on pleasantness. The result showed that there were significant differences between the five types of essential oils (*F* (4, 220) = 20.59, *p* < .001). Pairwise comparisons (post hoc) showed that in terms of pleasantness, sweet orange was significantly higher than the rosemary, lavender and mint, *ps* < .001, and there was no significant difference (*p* = .23) with lemon; lemon was significantly higher than rosemary, lavender and mint, *ps* < .001. No significant differences were found between the other pairs (all *ps* > .05).

Another repeated‐measures ANOVA was conducted to compare the effect of the five different scents on relaxation. The result showed that there were significant differences between the five types of essential oils (*F* (4, 220) = 15.40, *p* < .001). Pairwise comparisons (post hoc) showed that the results for sweet orange were significantly higher than those for rosemary, lavender and mint, *ps* < .001. Lemon was significantly higher than the rosemary, lavender and mint, *ps* < .001. Rosemary was significantly higher than lavender (*p* < .05). No significant differences were found between the other pairs (all *ps* > .05).

## CONCLUSION

The results showed that sweet orange essential oil was superior to the other four essential oils in terms of pleasantness and relaxation. Therefore, it was selected as the olfactory stimulus for the main study.

## MAIN STUDY

The purpose of this experiment is to examine whether ambient aromas of orange essential oil can improve the academic emotions of middle school students in a practical educational environment.

## METHODS

### Experiment design

A field experiment was conducted with pre‐ and post‐test experimental design upon subjects in three parallel classes in grade 2 of a junior school in China. The classroom specifications of the three classes were the same (length 9 m, width 6 m and height 3 m) and the floors, lighting, desks, chairs and other environmental conditions were basically the same. Two classes were used as the experimental subjects (in which scent diffusers containing diluted sweet orange essential oil were placed), and the other class was used as the control (in which scent diffusers containing purified water were placed). In order to explore the adaptive influence of smell, one experimental class was perfumed every day (experimental class 1) and the other experimental class was perfumed every other day (experimental class 2).

All procedures performed in studies involving human participants were in accordance with the ethical standards of the Shandong Provincial Academic Ethics Committee and with the 1964 Helsinki Declaration and its later amendments or comparable ethical standards.

All subjects and their parents received informed consent and agreed to participate in the study.

### Participants

Using the cluster random sampling method, three classes were randomly selected from grade two of a junior middle school, and then randomly divided into experimental class 1, experimental class 2 and the control class. There were 109 students (average age: 13.42 ± 0.56) in all three classes, including 56 boys and 53 girls. There were 36 students in experimental class 1, 37 students in experimental class 2 and 36 students in the control class. Prior to participation each volunteer completed a health questionnaire and these showed that all participants were in good health. None of the subjects had participated in the pre‐study. All participants and their parents were informed about the content of this study and gave their informed consent.

### Experimental materials and instruments

The emotion‐inducing materials are purified water and sweet orange essential oil (diluted to 3%). The instrument used in the experiment is a Smokeless and Flameless Aromatherapy Reed Diffuser (a 300 ml porcelain vase with aromatherapy cane and dried flowers). This apparatus relies on the natural volatilisation of the essential oil to achieve its purpose.

### Measurements

For the evaluation of academic emotions the Adolescent Academic Emotion Questionnaire (Dong & Yu, 2007) was used. The Questionnaire included a total of four subscales: positive high‐arousal academic emotion (pride, joy and hope), positive low‐arousal academic emotion (relaxation, calmness and satisfaction), negative high‐arousal academic emotion (anxiety, shame and anger) and negative low‐arousal academic emotion (boredom, helplessness, depression and upset). The Cronbach's coefficients of the four subscales were 0.81, 0.84, 0.83 and 0.89, respectively.

### Procedure

First, all the participants were pre‐tested and filled in the “Adolescent Academic Emotion Questionnaire.” Second, in the experimental classes, four diffusers filled with diluted sweet orange essential oil per class were used, and in the control class, four diffusers filled with pure water were used according to the experimental design. The essential oil solution or pure water were added every 10 days on average. All the participants filled out the “Adolescent Academic Emotion Questionnaire” again 8 weeks later. The whole experiment took about 8 weeks in the winter of 2019.

### Data analysis

Seven participants who answered the questionnaire incompletely were excluded, including three in experimental class 1, 3 in experimental class 2 and 1 in the control class. The remaining 102 were entered into the data statistics (33 in experimental class 1, 34 in experimental class 2 and 35 in the control class). Finally, SPSS 19.0 was used to collate and analyse the data.

## RESULTS

### Comparative analysis of pre‐test scores of academic emotions between experimental classes and the control class

Analysis of the pre‐test ratings indicated no differences between the three classes on any of the mood variables prior to the experimental session: Pride, *F*(2,101) = 0.11; Joy, *F*(2,101) = 0.58; Hope, *F*(2,101) = 2.27; Positive high‐arousal academic emotion, *F*(2,101) = 0.75; Relaxation, *F*(2,101) = 0.14; Calmness, *F*(2,101) = 0.89; Satisfaction, *F*(2,101) = 0.87; Positive low‐arousal academic emotion, *F*(2,101) = 1.13; Anxiety, *F*(2,101) = 0.68; Shame, *F*(2,101) = 1.98; Anger, *F*(2,101) = 2.80; Negative high‐arousal academic emotion, *F*(2,101) = 0.93; Boredom, *F*(2,101) = 0.31; Helplessness, *F*(2,101) = 1.72; Depression, *F*(2,101) = 0.01; Upset, *F*(2,101) = 0.16; and Negative low‐arousal academic emotion, *F*(2,101) = 0.99; *ps* > .05.

### Analyses compared post‐test minus pre‐test change in academic emotion scores between experimental class and control class

In order to test the change in the participants' academic emotions before and after the experiment, the change in academic emotions' scores (CS) were obtained by subtracting the pre‐test score for each participants' academic emotions from their post‐test score. The higher the CS in positive academic emotions, the higher the positive academic emotions after the experiment. The lower the CS in negative academic emotions, the lower the negative academic emotions after the experiment. One‐way ANOVA was subsequently conducted with the experimental and control class as independent factors, CS as the dependent variable and the results are shown in Table 2.

**TABLE 2:** Analysis of academic emotions' scores (CS) between experimental class and control class (*M ± SD*)

	Experimental class 1(N = 33)	Experimental class 2 (N = 34)	control class (N = 35)	F	p
Pride	1.45 ± 4.14	−0.47 ± 7.91	−1.60 ± 6.31	2.04	.14
Joy	1.88 ± 3.92	−0.82 ± 5.43	−6.23 ± 7.78	7.53	.001
Hope	−0.75 ± 3.15	−0.94 ± 2.99	−3.34 ± 5.02	4.72	.01
Positive high‐arousal academic emotion	−1.25 ± 7.35	−1.40 ± 11.43	−11.77 ± 16.89	7.25	.001
Satisfaction	2.97 ± 3.89	4.03 ± 3.72	2.77 ± 4.94	0.9	.41
Calmness	−1.56 ± 10.11	0.58 ± 3.19	−1.65 ± 4.81	1.18	.31
Relaxation	0.75 ± 4.13	1.06 ± 4.31	−2.24 ± 4.99	5.45	.006
Positive low‐arousal academic emotion	1.62 ± 14.52±	5.87 ± 8.64	−1.06 ± 13.06	2.61	.08
Anxiety	−2.70 ± 5.74	−2.21 ± 5.53	−1.46 ± 5.31	0.44	.65
Shame	−2.91 ± 3.25	−2.94 ± 3.69	−1.24 ± 4.08	2.34	.10
Anger	−7.06 ± 10.27	−3.28 ± 4.17	−2.47 ± 4.17	4.26	.02
Negative high‐arousal academic emotion	−12.87 ± 13.15	−8.56 ± 7.49	−5.46 ± 9.76	4.17	.02
Boredom	−3.00 ± 4.64	−3.96 ± 5.64	−1.22 ± 8.03	1.46	.24
Helplessness	−1.06 ± 3.60	−1.62 ± 3.82	0.51 ± 4.04	2.88	.06
Depression	−0.97 ± 4.57	−1.52 ± 4.22	−2.59 ± 3.69	1.70	.19
Upset	−0.32 ± 3.09	−1.09 ± 3.00	−0.94 ± 4.02	0.45	.64
Negative low‐arousal academic emotion	−5.30 ± 11.98	−7.12 ± 10.24	−4.39 ± 15.54	0.32	.73

Table 2 showed that in terms of joy, hope, positive high‐arousal academic emotion and relaxation, the differences among the three classes were significant (the effects were: *η* ^*2*^ = .18, *η* ^*2*^ = .12, *η* ^*2*^ = .16, *η* ^*2*^ = .17), *ps* < .05. In terms of anger and negative high‐arousal academic emotion, the differences of the three classes were significant (the effects were: *η* ^*2*^ = .09, *η* ^*2*^ = .18), *ps* < .05; There were no significant differences in other aspects of academic emotion.

Multiple comparison (LSD) found that in terms of joy, hope, positive high‐arousal academic emotions and relaxation, experimental classes 1 and class 2 were significantly higher than the control class (all *ps* < .01). In terms of anger, experimental class 1 was significantly lower than experimental class 2 (*p* = .03) and the control class (*p* = .007). In terms of negative high‐arousal academic emotions, experimental class 1 was significantly lower than the control class (*p* = .005).

## DISCUSSION

The aim of this research was to investigate the influence of ambient aroma on middle school students' academic emotions in a practical educational environment. Comparisons of the change in ratings from baseline to post‐test revealed that compared to the control class, joy, hope, positive high‐arousal academic emotion and relaxation in the ambient aroma condition were significantly higher, and angry and negative high‐arousal academic emotion in it were significantly lower. To our knowledge, this is the first study to examine the long‐term effects of aromatherapy with orange oil on students' academic emotions.

One finding of the present study was that positive academic emotion (except satisfaction) in the control class decreased significantly over time, and some positive academic emotion (e.g., joy, hope and positive high‐arousal emotion) also decreased in the experimental class. This result is supported by a recent study by Liu (2020), who found that positive academic emotions of middle school students decreased over time after a 3‐month follow‐up measurement. Therefore, the decline of positive academic emotion over time may be a common phenomenon. The reason may be that the interest and enthusiasm of students for school life decreases as the length of study increases and as homework and exam pressure increase. However, our results also found that after aromatherapy intervention the positive academic emotion of the experimental subjects decreased less than that of the control class, and some positive academic emotion (e.g., satisfaction, relaxation and positive low‐arousal academic emotion) had an increasing trend. These results further indicated that sweet orange essential oil could alleviate the decrease in some academic emotions or directly promote the increase of some positive academic emotions in the experimental group.

Another interesting result was that the negative valence variables declined over time in all groups. This result is inconsistent with existing studies. For example, Liu (2020) found that the negative academic emotion of middle school students increased over time. The result of the present study may be because the students were about to have a holiday when the second questionnaire was taken. The anticipation of a holiday might reduce their negative academic emotions. All the negative valence variables declined over time in all groups, but the decline for anger, negative high‐arousal academic emotion and helplessness were greater in the aroma groups. This result indicated that the aroma of orange essential oil played a role in reducing these negative academic emotions.

Another feature of the present study is to explore the effect of aromatherapy on the academic emotions of the students over a period of up to 8 weeks, and it was found that chronic aromatherapy could prevent the decline of their academic emotion over this period. This study demonstrates the long‐term effects of aromatherapy on students' academic emotions. Its results were supported by other studies, for example, a 4‐week study of aromatherapy interventions found that aromatherapy massage improved personal mood and quality of life in patients on chronic haemodialysis (Mohammadpourhodki et al., 2021).

Furthermore, aromas of orange essential oil were shown to reduce anxiety in patients waiting at a clinic (Eguchi et al., 2016; Fenko & Loock, 2014; Imura et al., 2006), to improve depression in patients (Imura et al., 2006), and to improve individual satisfaction in healthy adults (Moss et al., 2003). This study investigated the effect of ambient aroma on middle school students' academic emotions in their actual educational environment, and it found that the aroma of orange essential oil could improve their academic emotions. The results of this study extend the research scope of aromatherapy from emotions in medical environments to education and from general emotional problems to academic emotions.

The results of this study were also supported by brain science research. Odour and emotional brain studies have found that smells have the same effects on the brain as emotions, and that a smell (especially aroma) stimulus may activate the insula, anterior cingulate and orbitofrontal cortex, which are the areas closely related to emotions (Soudrya et al., 2011). In this study, the effect of aromatic odours on academic emotional improvement may also be due to the fact that teenagers are more sensitive to odours (Larsson et al., 2009). For example, one study found that performances by schoolchildren, aged 9–11 years, on working memory tasks were significantly better in the Rosemary aroma condition than in no aroma (Moss et al., 2017).

In addition, this study also found that there were no significant differences in terms of positive academic emotions and negative ones (except for anger) between experimental classes 1 and class 2. The results indicated that there was no olfactory adaptability problem towards the aroma in experimental class 1. This may be because students were not exposed continuously, as they left the classroom before 21:00 and arrived at 7:00, they also had extracurricular activities and physical education. This result indicates that long‐term use of aromatherapy in classrooms is beneficial to improve students' academic emotions.

## CONCLUSIONS

The findings revealed that the middle school students preferred the aroma of sweet orange essential oil. Also, the ambient aroma of this substance could mitigate the reduction of positive academic emotions and improve negative academic emotions in the studied school students over time. The study provides new ideas for schools to create a positive educational environment.
